# Critical metabolic pathways and genes cooperate for epoxy fatty acid-enriched oil production in developing seeds of *Vernonia galamensis*, an industrial oleaginous plant

**DOI:** 10.1186/s13068-022-02120-2

**Published:** 2022-02-25

**Authors:** Yan Sun, Baoling Liu, Jinai Xue, Xiaodan Wang, Hongli Cui, Runzhi Li, Xiaoyun Jia

**Affiliations:** Institute of Molecular Agriculture and Bioenergy, Shanxi Agricultural University, Taigu, Jinzhong, China

**Keywords:** Epoxy fatty acid (EFA), *Vernonia galamensis*, Transcriptome, Triacylglycerol, Industrial oil, Oilseed

## Abstract

**Background:**

*Vernonia galamensis* native to Africa is an annual oleaginous plant of Asteraceae family. As a newly established industrial oil crop, this plant produces high level (> 70%) of vernolic acid (*cis*-12-epoxyoctadeca-*cis*-9-enoic acid), which is an unusual epoxy fatty acid (EFA) with multiple industrial applications. Here, transcriptome analysis and fatty acid profiling from developing *V. galamensis* seeds were integrated to uncover the critical metabolic pathways responsible for high EFA accumulation, aiming to identify the target genes that could be used in the biotechnological production of high-value oils.

**Results:**

Based on oil accumulation dynamics of *V. galamensis* seeds, we harvested seed samples from three stages (17, 38, and 45 days after pollination, DAP) representing the initial, fast and final EFA accumulation phases, and one mixed sample from different tissues for RNA-sequencing, with three biological replicates for each sample. Using Illumina platform, we have generated a total of 265 million raw cDNA reads. After filtering process, de novo assembly of clean reads yielded 67,114 unigenes with an N50 length of 1316 nt. Functional annotation resulted in the identification of almost all genes involved in diverse lipid-metabolic pathways, including the novel fatty acid desaturase/epoxygenase, diacylglycerol acyltransferases, and phospholipid:diacylglycerol acyltransferases. Expression profiling revealed that various genes associated with acyl editing, fatty acid β-oxidation, triacylglycerol assembly and oil-body formation had greater expression levels at middle developmental stage (38 DAP), which were consistent with the fast accumulation of EFA in *V. galamensis* developing seed, these genes were detected to play fundamental roles in EFA production. In addition, we isolated some transcription factors (such as WRI1, FUS3 and ABI4), which putatively regulated the production of *V. galamensis* seed oils. The transient expression of the selected genes resulted in a synergistic increase of EFA-enriched TAG accumulation in tobacco leaves. Transcriptome data were further confirmed by quantitative real-time PCR for twelve key genes in EFA biosynthesis. Finally, a comprehensive network for high EFA accumulation in *V. galamensis* seed was established.

**Conclusions:**

Our findings provide new insights into molecular mechanisms underlying the natural epoxy oil production in *V. galamensis.* A set of genes identified here could be used as the targets to develop other oilseeds highly accumulating valued epoxy oils for commercial production.

**Supplementary Information:**

The online version contains supplementary material available at 10.1186/s13068-022-02120-2.

## Background

Plant oils are the most energy-dense compounds and major carbon available from nature, providing essential nutrients for human and also petroleum alternatives in the industry [1]. For producing oleochemicals such as plasticizers, adhesives, soaps, coatings, paints, lubricants and polymers, plant oils have to be chemically modified. One of the most high-valued modifications is to introduce an additional epoxy group [2]. Currently, the major sources of epoxy oils are produced by chemical epoxidation of highly unsaturated oils, such as soybean and linseed oils. However, chemical synthesis of epoxy group is a relatively expensive process, accompanied by emission of volatile organic compounds, which causes secondary pollution. Compared to the artificial products, natural epoxy oils represent the inexpensive, environmental-friendly and renewable feedstocks that could replace petrochemicals [3, 4].

For many years, natural epoxy oils were only found in some wild plants from Asteraceae and Euphorbiaceae species, including *Vernonia galamensis*, *Vernonia anthelmintica*, *Stokesia laevis*, *Crepis palaestina* and *Euphorbia lagascae* [5]*.* Notably, *V. galamensis* is an annual herb native to Africa, which contains the highest level of epoxy oil (about 40%), and thus is considered to be a good model for the study of epoxy oil metabolism [6]. In fact, the industrial value of natural epoxy oil arises from its high content of vernolic acid (*cis*-12-epoxy-octadeca-*cis*-9-enoic acid), which is an unusual epoxy fatty acid (EFA) that comprises > 70% of *V. galamensis* seed oil [7]. Like other plants able to produce EFA-enriched oils, large-scale cultivation of *V. galamensis* is limited by its poor agronomic traits [8]. Alternatively, creating genetically modified plant has been a promising strategy for commercial production of natural epoxy oil production [9]. As such, identification of genes for synthesis of EFA is one of the great challenges for such genetic engineering. However, the physiological and molecular mechanisms underlying the accumulation of EFA-enriched oil in *V. galamensis* seed and other source plants remain unknown.

It is now widely recognized that EFA is catalyzed by a functional variant of Δ^12^-fatty acid desaturase (FAD2), which is termed as epoxygenase [10]. So far, genes encoding epoxygenase have been isolated from *C. palaestina* (*Cpal2*) and *S. laevis* (*SlEPX*) [10, 11]. Transgenic expression of *Cpal2* in Arabidopsis has successfully yielded the synthesis of unusual EFA, but its content (6.2%) is very low [11]. Yu et al. evidenced that acyl-CoA:diacylglycerol acyltransferases (DGATs) from *V*. *galamensis* have strong preference for EFA-containing substrates, thus promoting the storage of large amounts of EFA into triacylglycerol (TAG) [12]. Simultaneous co-expression of *SlEPX* and *V. galamensis DGAT2* (*VgDGAT2*) in soybean seeds could result in EFA content of 25.8%, representing the maximum level achieved in engineered plants [13]. Despite that, transgenic experiments still fail to achieve high yields of desired EFA. It seems that additional factors may influence its normal accumulation in transgenic plants. Indeed, abnormally large amounts of EFA are esterified to PC (membrane lipids) in these engineered plants [13], while EFA in PC is very low in native source plants, where most of EFA is stored in TAG. Similar phenomena were also reported for transgenic production of other unusual fatty acids (FAs), such as hydroxy and conjugated FAs [14, 15]. Excessive accumulation of unusual EFA in membrane lipid often causes detrimental phenotype for transformed tissues. These results indicate that common oilseeds may lack specialized enzymes which can transfer the unusual EFA from the site on PC to their storage TAG [16, 17].

Undoubtedly, the production of high levels of EFA in engineered oilseeds will need the introduction of other genes involved in EFA metabolism, especially the specialized enzymes as-yet-unidentified from high-EFA accumulators. To facilitate this effort, we analyzed the FA compositions from developing *V. galamensis* seeds and generated the first assembly of *V. galamensis* transcriptome. As a result, nearly all genes involved in the processes of EFA metabolism, in particular, fatty acid modification, fatty acid β-oxidation, acyl editing, TAG storage, oil-body formation and transcription regulators were identified. Transient expression assays of several selected genes for enhancing EFA-enriched TAG accumulation in tobacco leaves were also performed. We further established a network for dissecting metabolic pathways involved in EFA and TAG biosynthesis based on the annotated transcriptome sequences and gene expression profiles. To the best of our knowledge, this study is the first report on analyzing the transcriptome data in oilseeds which produce EFA-enriched oils. The genes identified from *V. galamensis* seeds will provide valuable resources for reprogramming natural epoxy oil in current established oilseed crops to commercially produce such valued industrial oil in the future.

## Methods

### Plant materials and fatty acid analysis

Seeds of *V. galamensis* were obtained from National Infrastructure for Crop Germplasm Resources. In this study, *V. galamensis* plants were grown under normal growth condition at the experimental station of Shanxi Agricultural University, Taigu, China. Leaf, stem, and root tissues were harvested from 5-week-old plants. Flowers were collected at the full bloom stage, and the sampling time was recorded as 0 days after pollination (0 DAP). Developing seed samples were harvested at 10, 17, 24, 31, 38, 45, and 52 DAP, respectively. All samples were frozen in liquid nitrogen, and stored at − 80 ℃ until further use. The oil content and fatty acid composition were determined using the method described in our previous work [18]. Briefly, approximately 20 mg of powder sample was homogenized in 1 mL of chloroform and methanol (v:v = 2:1) containing 2 mg of tri-heptadecanoin (tri-C17:0) as the internal standard, and then methylated using 2 mL of methanolic sulfuric acid at 80 °C for 2 h. The resulting FA methyl esters (FAMEs) were isolated by *n*-hexane, and then briefly dried with nitrogen. The FAMEs were suspended in 1.5 mL of dichloromethane, and measured by Gas Chromatography (Agilent 7890B) [18].

### RNA extraction, library construction and sequencing

For transcriptome sequencing, total RNA isolated from different tissues (root, stem, leaf and flower) of *V. galamensis* was equally mixed together to form a mixed sample (T01), which was used to construct cDNA library. Importantly, seed samples of three representative stages of *V. galamensis* seed development at 17 DAP (T02), 38 DAP (T03), and 45 DAP (T04) were harvested, respectively, for cDNA library construction. Total RNA from each sample was isolated using Trizol Reagent (Shenggong, China) following the manufacturer’s protocol. After checking the quality and quantity with NanoDrop spectrophotometer (NanoDrop Technologies, USA) and Agilent 2100 Bioanalyzer (Agilent Technologies, USA), mRNA was enriched using oligo (dT) beads and then broken into short pieces by random shearing. We further used these fragments as templates for first and second strand cDNA synthesis. Obtained cDNA fragments were then processed with end repair, Illumina’s paired-end adapter ligation, size range selection, purification and PCR amplification. Finally, 12 paired-end cDNA libraries with three biological replicates in each indicated sample were constructed and then sequenced on flow cell using Illumina HiSeq™ 2500 system in Biomarker Technology Corporation (Beijing, China).

### Transcriptome assembly

After sequencing, RNA-Seq data were generated in the form of Fastq. The raw data were then processed by removing the reads with more than 5% of unknown nucleotides, the reads containing more than 20% of low-quality bases (quality score less than 30), empty reads and adaptor sequences. The filtered reads from all samples were then merged and de novo assembled using Trinity assembler with default parameters and ‘K-mer size = 25’ [19]. Trinity program included three independent processing modules. First, clean reads were extended into longer contigs by merging overlapping regions. Second, contigs from the same transcript were further connected based on paired-end match information. Finally, the sequences which could not be extended on either end were screened by redundancy removal tool. The unique consensus sequences were defined as unigenes, which represented the reference data set for *V. galamensis* transcriptome.

### Sequence annotation and estimation of differentially expressed genes

Gene functions were annotated by BLASTX searches (*E* value < 1E-5) against public databases, including NCBI non-redundant (Nr), Swiss-Prot, Kyoto Encyclopedia of Genes and Genomes (KEGG), COG and KOG. Gene ontology (GO) terms were assigned based on Nr annotations using Blast2GO program, and GO tree was illustrated by WEGO tool [20]. The expression abundance of each unigene was counted and normalized using FPKM method, which could eliminate the effects of gene length and sequencing depth [21]. The quantitative FPKM values could be directly used to calculate the transcriptional difference between different samples. Differentially expressed genes (DEGs) were determined using the Bioconductor package (EBSeq), in which Benjamini–Hochberg model was selected to adjust for multiple tests. The threshold of DEG was defined with statistically significant false discovery rate (FDR) of at most 0.001 and fold change of at least 2 [22]. Functional enrichment analyses of DEGs in GO terms were performed using Fisher’s exact test. Hyper geometric test was applied to determine DEGs significantly enriched in KEGG pathway [23].

### Transient expression of the target genes in *Nicotiana benthamiana*

The coding sequence (ORF) of each candidate gene for examination was cloned by RT-PCR using high-fidelity PCR kit. The primers were designed based on the target transcripts annotated. Each ORF of the candidate genes was cloned into the plant expression vector pCAMBIA1303 and subsequently introduced into *Agrobacterium tumefaciens* GV3101. Transient overexpression of the selected genes individually or in combination in *N. benthamiana* leaf tissues was carried out as described in our previous study [18]. For coexpression assay, *A. tumefaciens* cultures containing the gene coding for *V. galamensis* epoxygenase and other target genes were mixed until the final OD600 of 0.25 prior to infiltration. The empty-vector (EV) infected leaves were used as blank controls. Leaf samples were collected 4 days after infiltration and then analyzed for fatty acid composition. The selected gene sequences for such examination are listed in Additional file 1.

### Quantitative real-time PCR analysis

The quantitative Real-Time PCR (qRT-PCR) was carried out in CFX96 Real-Time PCR system (Bio-Rad, USA) with three replicates. Following reaction conditions were applied: 95 °C for 10 min, 40 cycles of 95 °C for 15 s, 60 °C for 1 min, 72 °C for 20 s. The melting curve was analyzed after the final cycle, ramping from 65 °C to 95 °C with increment of 0.5 °C/5 s. The normalized relative expression was calculated using 2^−ΔΔCt^ method. *V. galamensis actin* gene was employed as internal reference. Gene-specific primers were listed in Additional file 2.

## Results

### Dynamic patterns of oil content and fatty acid composition in developing *V. galamensis* seeds

**Fig. 1:**
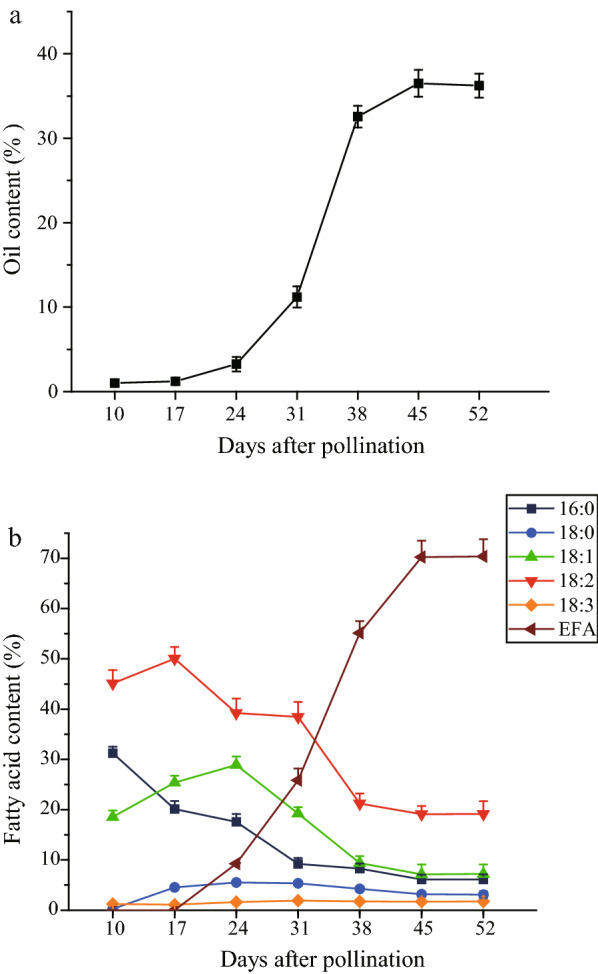
Lipid content and composition dynamics across the developmental stages of *V. galamensis* seeds. **a** The lipid content of developing *V. galamensis* seeds. **b** The composition of the six major fatty acids in developing *V. galamensis* seeds (mean ± SD, *n* = 3). Seeds were harvested at 10 days after pollination (DAP, immature stage), and then every 7 days until 52 DAP (mature stage)

To select the optimal stages for transcriptome sequencing, the oil accumulation patterns during *V. galamensis* seed development were analyzed (Fig. 1). The results showed that only a little oil was produced in the developing seeds at 10 and 17 DAP (1.77% and 1.84%, respectively), but a most noticeable increase in seed oil content occurred from 24 to 38 DAP. Thereafter, the growth rate of oil accumulation changed gradually till 45 DAP when the maximum oil level (35.8%) was reached (Fig. 1a). We further monitored the dynamic transition for fatty acid components in *V. galamensis* seed oils, including vernolic acid (EFA), palmitic acid (16:0), stearic acid (18:0), oleic acid (18:1), linoleic acid (18:2) and linolenic acid (18:3). On the whole, the percentage of EFA showed a similar increasing trend with total oil accumulation in developing *V. galamensis* seeds. Accompanying the accumulation of EFA, oleic acid and linoleic acid contents were largely decreased. Palmitic acid level gradually declined during seed development. In addition, there was little change in stearic acid and linolenic acid which make up a lower relative proportion in developing seeds (Fig. 1b).

### Transcriptome sequencing and de novo assembly

**Fig. 2:**
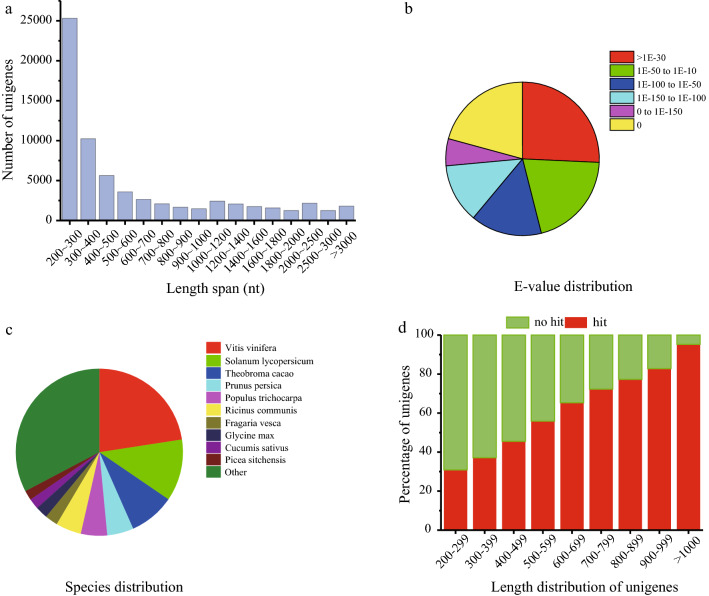
Characteristics of unigenes in *V. galamensis*. **a** Size distribution of all assembled unigenes. **b** E-value distribution of best BLASTX hits for each unigene. **c** Species distribution of top BLAST hits for each unigene in Nr database. **d** Length of unigenes with hits or no hits

To identify candidate genes associated with EFA biosynthesis and metabolism, we prepared a mixed sample (T01) and three seed samples representing three developing stages at 17 (T02), 38 (T03), and 45 DAP (T04), which are consistent with the initial, fast and final stages of EFA accumulation, for transcriptome sequencing. Twelve cDNA libraries considering three biological replicates for each sample generated around 265 million raw reads. After quality-control processing, a total of 77.6 Gb nucleotide bases were obtained. The average Q30 base ratio (sequencing quality value) and GC percentage for each library was 92.75 and 44.94%, respectively (Additional file 3). These reads were then de novo assembled by Trinity toolkit, resulting in 67,114 unigenes with a mean length of 729.96 nt (nucleotides) and N50 length of 1316 nt (Additional file 4). The assembled unigenes mainly ranged from 200 to 2000 nt, and unigene numbers had a trend of gradual decrease as the length of unigene increased. Of the unigenes, 25,321 (37.73%) sequences had a shorter length range between 200 to 300 nt, 9132 (13.61%) transcripts were between 1000 to 2000 nt, and 5261 (7.84%) sequences were longer than 2000 nt in length (Fig. 2a).

### Functional annotation, classification and DEG analysis

Based on sequence similarity search, 35,680 (53.16%) unigenes were functionally annotated in at least one public database, whereas the function of rest sequences (31,434) remained unknown (Additional file 5). The *E* value distribution of BLASTX hits showed that 38% of unigenes shared strong homology to previously reported sequences (< 1.0E−100) (Fig. 2b). Unigenes from *V. galamensis* had the most similarities with genes from *Vitis vinifera* (7981), followed by *Solanum lycopersicum* (4183) and *Theobroma cacao* (3088) (Fig. 2c). Overall, the longer sequences were more easily annotated with BLAST hits (Fig. 2d).

Based on Nr annotation, a total of 26,882 (40.05%) unigenes could be assigned to three main GO categories, including biological process (21,364 unigenes, 31.83%), molecular function (21,031 unigenes, 31.34%) and cellular component (17,749 unigenes, 26.45%). Within the biological process, metabolic process and cellular process represented the dominant GO terms. In category of cellular component, cell part and cell were the two top sub-categories. The terms related to binding and catalytic activity were most abundant in molecular function (Additional file 6). Eukaryotic Orthologous Groups (KOG) analysis showed that the matched 28,896 unigenes were divided into 26 function classes, with the signal transduction mechanism (2406 unigenes) as the largest group (Additional file 6). From KEGG system, 8371 unigenes were annotated to 118 metabolic pathways (Additional file 7). The most predominant pathways were ribosome (ko03010, 771), followed by oxidative phosphorylation (ko00190, 350) and protein processing in endoplasmic reticulum (ko04141, 317). Moreover, we also annotated several regulation networks of lipid accumulation, including fatty acid metabolism (ko00071, 101), biosynthesis of unsaturated fatty acids (ko01040, 77), glycerolipid metabolism (ko005610, 72) and alpha-linolenic acid metabolism (ko00592, 21).

**Fig. 3:**
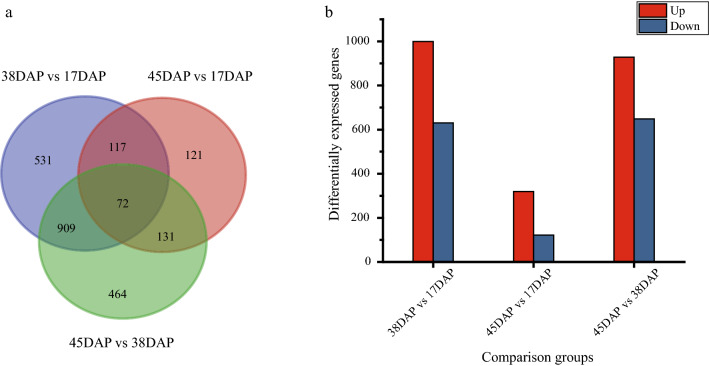
Distribution of differentially expressed genes (DEGs) in *V. galamensis* at different developmental stages. **a** Number of DEGs in different comparisons. **b** Venn diagram of DEGs in different comparisons: 38DAP vs 17DAP, 45DAP vs 38DAP, and 45DAP vs 17DAP

In our study, FPKM value was used to quantify the expression level of each unigene. Gene expression levels were further analyzed by pairwise comparisons across the different developmental seed stages (initial, fast and final phases of EFA accumulation) to identify differentially expressed genes (DEGs). Our criteria led to the identification of 2345 DEGs across three developing samples. There were 531, 464, and 121 genes that were differentially expressed specifically in the group of 38 DAP vs 17 DAP, 45 DAP vs 38 DAP, and 45 DAP vs 17 DAP, respectively (Fig. 3a). In the comparison of 38 DAP vs 17 DAP, there existed the largest number of DEGs (1629) and up-regulated genes (999). Only 441 DEGs were identified in 45 DAP vs 17 DAP, including 138 down- and 303 up-regulated genes (Fig. 3b). The results showed that a more remarkable change in gene expression occurred in 38 DAP vs 17 DAP, as compared to 45 DAP vs 38 DAP. It was noteworthy that the number of up-regulated genes outnumbered the down-regulated genes in all comparison groups.

To explore the functional differences among DEGs in developing *V. galamensis* seeds, GO and KEGG enrichment analyses were conducted. First, 651 annotated DEGs were assigned to 42 functional subgroups within three main GO categories, implying that these DEGs were functionally involved in diverse physiological processes (Additional file 8). Among these unigenes, the most enriched terms were those involved in regulatory activity for DNA replication, G2/M transition of mitotic cell cycle, and histone phosphorylation. Furthermore, many DEGs were related with the process of fatty acid hydrolysis, fatty acid alpha-oxidation and unsaturated fatty acid biosynthesis (Additional file 9). We highlighted that these enzymes may play critical roles in determining the fatty acid composition in *V. galamensis* seeds. Furthermore, 495 DEGs were mapped to 87 KEGG pathways. After pathway enrichment, we noticed that abundant DEGs were included in fatty acid biosynthesis, biosynthesis of unsaturated fatty acid, glycerolipid metabolism, glycosphingolipid biosynthesis, and sphingolipid metabolism, which were closely related to lipid accumulation in *V. galamensis* seed.

### Characterization of genes contributing to high EFA accumulation in *V. galamensis* seeds

**Fig. 4:**
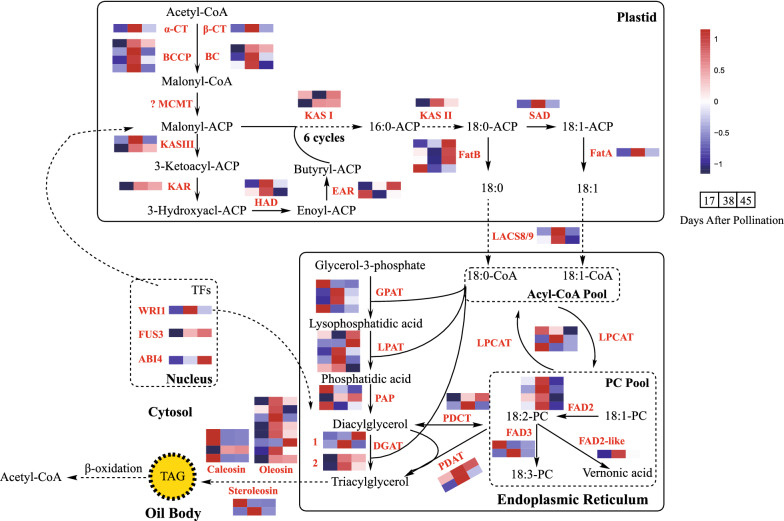
Proposed fatty acid and TAG biosynthetic pathways in *V. galamensis* seeds. The reconstructed pathway included the fatty acids de novo biosynthesis, fatty acid modification, triacylglycerol (TAG) assembly and storage process. The heatmap showed the FPKM value of each unigene in the initial, fast and final oil accumulation stages. Abbreviations are listed in Additional file 10

Although EFA is greatly enriched in *V. galamensis* seed oil, the molecular mechanism underlying its high accumulation is still unclear. The primary purpose of our study is to uncover crucial genes involved in the high accumulation of EFA. These EFA-related genes would be used in genetic manipulation for commercial production of industrial-valued epoxy oil in traditional oilseed crops. Based on functional annotations, we identified 116 unigenes relevant to epoxy oil metabolism (Additional file 10), including the previously reported VgDGAT1 and VgDGAT2 [13], further showing that transcriptome analysis is a powerful tool for mining genes of interest. We then fully characterized all critical steps and enzymes involved in EFA-enriched oil biosynthetic pathway (Fig. 4).

It is generally known that FA synthesis begins with the transformation of acetyl-CoA to molonyl-CoA catalyzed by rate-limiting acetyl-CoA carboxylase (EC: 6.4.1.2, ACCase), an enzyme complex composed of four subunits: alpha-carboxyltransferase (α-CT), beta-carboxyltransferase (β-CT), biotin carboxyl carrier protein (BCCP) and biotin carboxylase (BC). We detected nine unigenes encoding ACCase subunits (one for α-CT, one for β-CT, four for BCCP, and three for BC protein, respectively). The expression of these nine genes exhibited a coordinated pattern, with the highest expression level observed at 38 DAP. After synthesis of malonyl-CoA, an acyl carrier protein (ACP) was covalently linked to malnonyl group by malonyl-CoA:ACP transacylase (EC: 2.3.1.39, MCAT) to form malonyl-ACP, an important substrate for following acyl chain elongation. During FA elongation process, butyryl-ACP was initially generated after the first condensation cycle. This reaction was completed within the participation of four successive enzymes: beta-ketoacyl-ACP synthase III (EC: 2.3.1.180, KAS III), NADPH-dependent beta-ketoacyl-ACP reductase (EC: 1.1.1.100, KAR), 3-Hydroxyacyl-ACP dehydratase (EC: 4.2.1.159, HAD) and enoyl-ACP reductase (EC: 1.3.1.9, EAR). Butyryl-ACP was further elongated to C16-ACP during the following six cyclic reactions, each of which used the same enzymes as the former, but KAS III was replaced by KAS I (EC: 2.3.1.41). Whereas the last elongation of C16-ACP to C18-ACP required a third condensing enzyme (KAS II, EC: 2.3.1.179). We detected nearly all unigenes involved in the biosynthesis of C18-ACP, but MCAT was missed in our data, which may be due to its rare expression abundance in *V. galamensis*. Considering malonyl-ACP produced from MCAT was an important substrate for following acyl chain elongation, it is possible that other unknown genes may determine the transfer of malonyl moiety to ACP in this EFA-enriched plant. Most 18:0-ACP obtained from final elongation process could be desaturated by stearoyl-ACP desaturase (EC: 1.14.19.2, SAD). There are seven SAD family genes in Arabidopsis, and of them, AtFAB2 (At2g43710) has a primary role in dehydrogenation reaction. We found that there existed only one SAD gene (homolog of AtFAB2) in *V. galamensis* seed, with the highest expression at 38 DAP. The elongation of FA was terminated when acyl group was released by acyl-ACP thioesterase (EC: 3.1.2.14, Fat), yielding free FA molecular. Two *Fat* gene families, *FatA* with a preference for unsaturated 18:1-ACP and *FatB* showing marked activity on saturated FAs, were detected to have 1 and 4 members, respectively. The expression levels of *VgFatA* and *VgFatB* exhibited opposing trends, and only *VgFatA* expression was the highest at 38 DAP. These free FAs were ultimately linked to CoA by long chain acyl-CoA synthetase (EC: 6.2.1.3, LACS) and exported to endoplasmic reticulum (ER) for modification. In our data set, 22 unigenes encoding LACS were successfully annotated. Two unigenes, one for ER-localized LACS8 and one for plastid-localized LACS9, were deemed as DEGs throughout seed development.

**Fig. 5:**
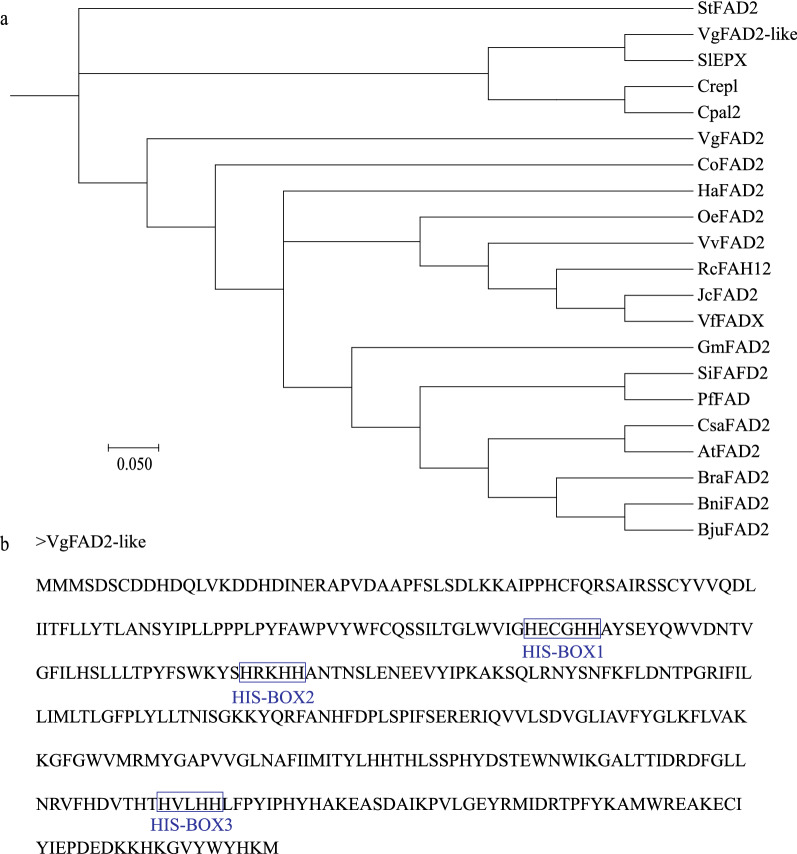
Characterization of FAD2-like protein in *V. galamensis*. **a** Phylogenetic comparison of VgFAD2-like, VgFAD2 and other selected plant FAD2 and divergent forms of FAD2 sequences. The tree was generated by ClustalW, Neighbor-Joining (NJ) method was adopted. Sequences used to construct the tree included *Arabidopsis thaliana* oleate desaturase AtFAD2 (NM_112047), *Ricinus communis* hydroxygenase RcFAH12 (U22378), *Vernicia fordii* conjugase VfFADX (AF525535), *Crepis palaestina* epoxygenase Cpal2 (Y16283), *Stokesia laevis* epoxygenase SlEPX (AY462108), *Crepis alpine* acetylenase Crepl (Y16285.2), *Vernonia galamensis* VgFAD2 (AF188264), *Glycine max* GmFAD2 (NP_001347010.1), *Olea europaea* OeFAD2 (QKI80086.1), *Camellia oleifera* CoFAD2 (QID89773.1), *Brassica nigra* BniFAD2 (ADJ58018.1), *Brassica rapa* BraFAD2 (XP_009146642.2), *Brassica juncea* BjuFAD2 (QGW48100.1), *Camelina sativa* CsaFAD2 (ADN10825.1), *Jatropha curcas* JcFAD2 (AEW43691.1), *Vitis vinifera* VvFAD2 (RVX12826.1), *Helianthus annuus* HaFAD2 (XP_035830434.1), *Perilla frutescens* PfFAD2 (APQ41587.1), *Sesamum indicum* SiFAD2 (XP_011075145.1) and *Solanum tuberosum* StFAD2 (XP_006365798.1). **b** The position of three conserved histidine boxes in FAD2-like protein sequence

In ER, most acyl-CoAs needed to be transferred to PC pools via lysophosphatidylcholine acyltransferase (EC: 2.3.1.23, LPCAT) for further desaturation or modification. This reaction and its reversible reacylation catalyzed by LPCAT was the key component of acyl editing. In general, oleic acid (C18:1) was channeled into PC, and then sequentially desaturated by ER-associated FAD2 and FAD3 to form C18:2 and C18:3. *V. galamensis* seed contained 70% EFA, and this modified fatty acid was controlled by a divergent form of FAD2. In our transcriptome, we detected four unigenes encoding oleate desaturases (FAD2). These four genes were expressed at a relatively steady level, and quickly dropped at the final seed developmental stage (45 DAP). We also discovered a novel FAD2 variant, which was designated VgFAD2-like. The FAD2-like enzyme was most closely related to Δ^12^ fatty acid epoxygenase identified from *S. laevis* (Fig. 5a). This enzyme contained the typical histidine box domains (Fig. 5b). In addition, *VgFAD2-like* gene showed bell-shaped expression pattern with a peak at 38 DAP seeds. The increased expression of this gene is coincided with the increased accumulation of EFA-containing TAG during *V. galamensis* seed development, suggesting that VgFAD2-like was likely a fatty acid epoxygenase in *V. galamensis*. Two *VgFAD3* genes were detected, and their expressions were very low, especially when seeds progressed to mid- and late-stages of development. After modification, PC releases FA into acyl-CoA pool through a constant deacylation reaction by LPCAT. Alternatively, PC can remove its phosphocholine head group and then degrade into DAG by phosphatidylcholine:diacylglycerol cholinephosphotransferase (PDCT). Phospholipid:diacylglycerol acyltransferase (PDAT) can also directly transport FA from PC into the *sn*-3 position of DAG to produce TAG, which is referred to as PC-mediated TAG assembly (Fig. 4). The generated FAs on CoA (including de novo synthesized, desaturated and modified FAs) were subsequently assembled into glycerol-3-phosphate (G3P) to form TAG, which is referred as the Kennedy pathway. In acyl editing pathway, we isolated one unigene of LPCAT1 and two unigenes of LPCAT2. Of them, only *LPCAT1* gene was highly expressed throughout all developmental stages. For Kennedy pathway-related enzymes, we found that there were four, five, three and four unigenes encoding GPAT, LPAT, PAP, and DGAT (including two DGAT1 and two DGAT2), respectively. Among those 16 unigenes in this pathway, 9 unigenes exhibited a high transcript level in *V. galamensis* seed, and five unigenes substantially up-regulated their expression level, including two *GPATs*, one *DGAT1* and two *DGAT2s* at 38 DAP seeds. For PC-mediated TAG synthesis, *VgPDAT1* was ubiquitously expressed in developing seeds at all stages, while *VgPDAT2* showed a linear rise during seed development up to 38 DAP, and then decreased to a low level at 45 DAP. However, the expression level of *VgPDCT* was negligible in *V. galamensis* seeds.

Once synthesized, TAG is storaged in the form of oil bodies which is stabilized by phospholipid-containing proteins, such as oleosins, caleosins and steroleosins. We identified eight unigenes encoding oleosin, four encoding caleosin and two encoding steroleosin, respectively. In *V. galamensis* seeds, expression levels of oleosin genes were the most abundant, followed by caleosins and steroleosins.

It is generally known that fatty acid β-oxidation is essential for catabolism of stored TAG in seed-oil storing plants. The suppression of the TAG-disassembling genes might be conducive to oil accumulation in oilseeds. Unexpectedly, we noticed the expression levels of multiple genes related to fatty acid β-oxidation, including acyl-CoA oxidase (ACX), enoyl-CoA hydratase (ECH), and 3-ketoacyl-CoA thiolase (KAT), displayed a significant up-regulation from 17 to 38 DAP (Additional file 11).

Finally, we examined the transcriptional profiles of certain transcription factors with potential roles in fatty acid biosynthesis and oil accumulation. WRINKLED1 (WRI1) is a master regulator for plant oil synthesis pathway. This TF belongs to the Apetala2 ethylene response element binding factor (AP2/EREB) family. We found that *VgWRI1* was quickly up-regulated in the early period of oil accumulation, then kept a relatively high expression in middle period, and finally declined in the late developing period in *V. galamensis* seed. These results supported a critical role for VgWRI1 in the early and middle period of *V. galamensis* fatty acid synthesis and oil accumulation. FUSCA3 (FUS3) and ABSCISIC ACID INSENSITIVE4 (ABI4), two other lipid-metabolism-related transcription factors, were highly expressed in the late period of oil accumulation in *V. galamensis* seeds. Except for these unigenes, we did not identify DEGs ecoding other TFs relevant to lipid metabolism, such as LEAFY COTYLEDON1 (LEC1), LEC2, and MYB89.

### Transient expression of the selected genes for enhancing EFA-enriched TAG accumulation in tobacco leaves

To determine whether the *VgFAD2-like* gene isolated from our transcriptome data has catalytic specificity for EFA formation, we transiently expressed this gene in tobacco leaves mediated by Agrobacterium infiltration. As expected, the presence of a large amount of EFA (8.2%) was detected by GC analysis, suggesting the potential role of VgFAD2-like enzyme in manipulation of high-valued expoy oil (Table 1). Besides, we also tested the effect of VgLPCAT1 and VgPDCT on EFA incorporation in TAG. Using tobacco transient assay system, coexpression of *VgLPCAT1* and *VgFAD2-like* gene resulted in further increased amounts of EFA compared to the individual expression of *VgFAD2-like* gene alone (Table 1). By contrast, there was no significant change in fatty acid composition for additional expression of *VgPDCT* gene compared to the *VgFAD2-like* alone (Table 1). These observations indicated that VgLPCAT rather than VgPDCT contributed greatly to EFA accumulation in the TAG, possibly due to the increased efficiency of EFA removal from PC. Previous study has found that the level of accumulation of EFA is often influenced by the availability of the substrate linoleic acid [11]. To test this factor, we first demonstrated that overexpression of *VgFAD2* can result in a significant increase in linoleic acid content. Coexpression of *VgFAD2* and *VgFAD2-like* gene produced further enhancement of EFA accumulation compared to the expression of *VgFAD2-like* gene alone (Table 1). In addition, we investigated whether coexpression of *VgFAD2-like* with other acyltransferase genes could increase the transfer of EFA from EFA-CoA into TAGs. As shown in Table 1, EFA accumulation increased significantly when *VgLPAT* was coexpressed along with *VgFAD2-like*, indicating that the VgLPAT may have substrate preference towards EFA. However, coexpression of *VgFAD2-like* and *VgGPAT* resulted in no further increase of EFA accumulation compared to the expression of *VgFAD2-like* alone, suggesting that VgGPAT may be unable to select EFA for TAG assembly. Taken together, the above-mentioned gene sets represent a likely promising resource for future metabolic engineering of EFA production in high-biomass plants such as tobacco and the commercial oilseed crops such as soybean.

**Table 1:** Fatty acid composition of *Nicotiana benthamiana* leaves transiently expressing the selected genes

Lines	Fatty acid composition (% of total)						
	16:0	18:0	18:1	18:2	18:3	20:0	EFA
Control	21.6 ± 0.3	7.3 ± 0.1	3.9 ± 0.1	16.4 ± 0.3	42.7 ± 0.7	7.1 ± 0.2	0
VgFAD2	23.9 ± 0.4	8.8 ± 0.2	6.1 ± 0.1	28.2 ± 1.1	24.3 ± 0.5	8.9 ± 0.1	0
VgFAD2-like	21.5 ± 0.5	7.9 ± 0.3	4.1 ± 0.1	17.5 ± 0.5	33.2 ± 0.9	5.8 ± 0.1	8.6 ± 0.2
VgFAD2-like + VgPDCT	22.3 ± 0.5	7.6 ± 0.1	3.7 ± 0.4	17.9 ± 0.6	32.4 ± 1.2	6.4 ± 0.5	8.2 ± 0.1
VgFAD2-like + VgLPCAT	21.4 ± 1.6	7.9 ± 0.9	4.3 ± 0.2	21.3 ± 0.9	22.5 ± 1.9	4 ± 0.3	19.4 ± 0.4
VgFAD2-like + VgFAD2	15.3 ± 0.3	9.6 ± 0.1	7.2 ± 0.1	12.6 ± 0.4	38.1 ± 1.2	3.8 ± 0.1	13.1 ± 0.6
VgFAD2-like + VgGPAT	21.3 ± 0.3	7.6 ± 0.1	3.7 ± 0.4	17.9 ± 0.6	32.4 ± 1.2	6.4 ± 0.5	7.7 ± 0.1
VgFAD2-like + VgLPAT	20.2 ± 0.6	8.1 ± 0.3	4.1 ± 0.2	19.3 ± 0.4	25.5 ± 1.9	6.1 ± 0.3	16.7 ± 0.5

Error bars ± SD, *n* = 3

### Validation of gene expression using quantitative real-time PCR

**Fig. 6:**
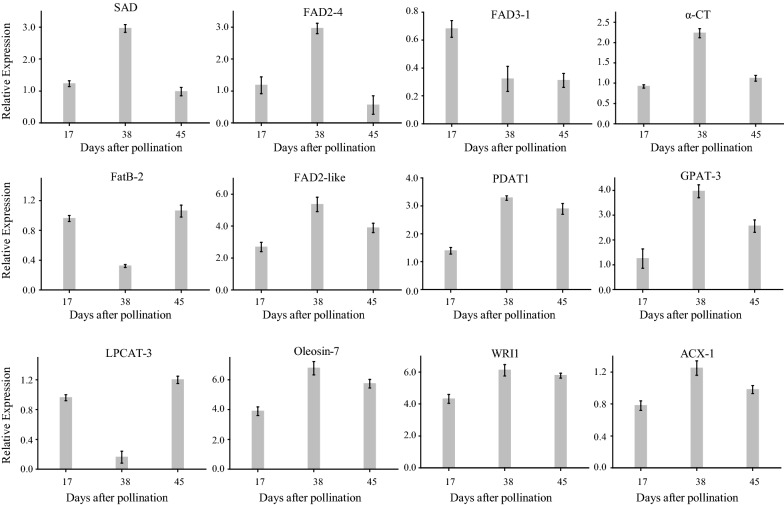
Relative expression of fatty acid synthesis genes in *V. galamensis* seeds at different developmental stages

To validate the RNA-seq data, twelve genes (*α-CT*, *FatB*, *SAD*, *FAD2*, *FAD2-like*, *FAD3*, *GPAT*, *LPCAT*, *PDAT*, *WRI1*, *Oleosin*, *ACX*) associated with lipid biosynthesis were analyzed by qRT-PCR. The results showed that the gene expression levels detected by qRT-PCR analysis are consistent with those obtained by transcriptome data (Fig. 6, Additional file 12), thus confirming that the data from RNA-Seq were reliable.

## Discussion

Epoxy fatty acid (EFA) is an important unusual fatty acid with broad industrial applications. In nature, only a limited number of wild plant species have the capacity to synthesize EFA in their seeds. However, the molecular mechanism of EFA accumulation in these source plants is yet to be explored. Due to the high EFA percentage in seed oil, *V. galamensis* has been recognized as a model plant for exploring the molecular mechanism and regulatory of EFA biosynthesis and accumulation. Given the commercialization of EFA is limited by its unfavorable agronomy status, the analysis of EFA metabolism in *V. galamensis* seed will provide potential genetic resources for sustainable production of EFA-enriched oils in other plants via genetic engineering.

With the focus on effective and reliable data, RNA-seq has been widely applied in the identification of target genes at whole-transcriptome level, especially in non-model species, including some oilseed crops for which reference genomes are not available [24]. By the aid of transcriptome analysis, Tian et al. successfully identified a list of important genes involved in unusual hydroxy fatty acid metabolism in *Hiptage benghalensis* seeds [25], which also verified the feasibility of our experiment design. In this study, for better understanding the EFA biosynthesis and regulation at the molecular level, transcriptome sequence was utilized for the identification of candidate genes contributing to high EFA accumulation and their expression profiles in developing *V. galamensis* seeds. First, changes in oil content and FA profiles during seed development were recorded (Fig. 1). Like the reports that oil accumulation often occurred at the mid-late stage of seed development in oilseed plants [23], the total lipids in *V. galamensis* seed rapidly accumulated between 31 and 38 DAP, reaching a peak at 45 DAP and then decreasing. At 52 DAP, the seed was mature and tended to stop growing. Intriguingly, the trend of EFA accumulation was in parallel with total lipid content, and this result was consistent with that reported by Li et al. [26]. Therefore, we selected three development stages of seed samples, which represented the initial, fast and final phases of EFA accumulation, respectively, for transcriptome analysis. To uncover more genes which are not exclusively expressed in developing seeds, we also sequenced a mixed sample prepared from RNA pool of different tissues. As a result, an assembly of 67,114 unigenes with a mean length of 729.96 nt was generated for *V. galamensis*, which were comparable to transcriptome data from other oilseed plants such as bitter melon [27], *Malania oleifera* [28], and *Camelina sativa* [29]. However, only 53.16% of unigenes identified here were functionally annotated. This relatively low rate might be due to the limited genomic information available for *V. galamensis*. In addition, non-annotated unigenes may be the consequence of having many shorter sequences, incomplete conserved domains or species-specific sequences.

Using linoleic acid (18:2)-PC as substrate, EFA was directly produced by FAD2-like enzyme (epoxygenase) at the *sn*-2 position of PC [13]. The linoleic acid content was high (over 38%) at initial seed development until 17 DAP. This quantity of linoleic acids seemed to be stored as ample substrates for further introduction of epoxy group. Except for EFA, linolenic acid (18:3) was also synthesized from linoleic acid via the desaturation catalyzed by FAD3 [30]. However, we observed that the linolenic acid content always remained at a relatively low level, while EFA was rapidly synthesized from 17 to 38 DAP. Accompanying the accumulation of EFA, transcript level of epoxygenase gene also increased steadily. However, the expression of *FAD3* genes kept low levels throughout seed development. Low activity of *FAD3* seemed to determine the production of EFA by blocking the flow of linoleic acid substrate into linolenic acids. These collective data may account for the relatively high content of EFA in developing *V. galamensis* seeds.

In plants, most free fatty acids need to be assembled into storage TAG. Only a small fraction can be bound to membrane. *V. galamensis* seed contained over 70% EFA, which was synthesized by Δ^12^ epoxygenase activity on linoleic acid bound to PC. Despite its main synthesis on PC, few EFA kept binding to PC in cells, but most of EFA was found to be stored in TAGs during the maturation of *V. galamensis* seeds. However, unusual fatty acids including EFA in the engineered plant seeds were mostly esterified to PC (membrane lipids), where they had limited reserves in TAGs [11]. These facts implied that there should exist an active channel from EFA synthesis on PC to its accumulation on TAGs in *V. galamensis* seeds. It is generally accepted that there are three pathways involved in the flux of newly generated FA from PC to TAG (Fig. 4). First, PC releases FA into acyl-CoA pool through a constant deacylation reaction by LPCAT, and thus the modified FA could enter Kennedy pathway for TAG assembly. Second, PC removes phosphocholine head group and degrades into DAG by PDCT, and the resulted DAG could be further utilized by DGAT to form TAG. Third, PC directly transfers FA into the *sn*-3 position of DAG to produce TAG by PDAT. To study the flux of EFA from PC to TAG, the above-mentioned lipid biosynthetic enzymes are of particular interest.

LPCAT enzymes play major roles in acyl editing, which include the incorporation of newly generated FA into PC (forward reaction), or transferring FA generated from PC to acyl-CoA pool through reversible acylation. Previous work has observed that the mutation of *LPCAT* genes in Arabidopsis resulted in a decrease of polyunsaturated fatty acid level [31]. In the case of unusual fatty acid, Lager et al. first studied the substrate specificity and selectivity of LPCAT enzyme in its reversible reaction [32]. By biochemical method, they found LPCAT enzymes from the hydroxy fatty acid-accumulating plants catalyzed the acylation *sn*-2 position of PC with great preference for hydroxy group, while LPCAT enzymes from Arabidopsis did not exhibit such selectivity. These results suggested an important role for LPCAT enzyme in removing desaturated and modified fatty acid from PC to acyl-CoA pool. Arabidopsis genome contained two *LPCAT* genes, *LPCAT1* (At1g12640) and *LPCAT2* (At1g63050). We have identified one unigene of *VgLPCAT1* (c9083.graph_c0) and two *VgLPCAT2* unigenes (c33748.graph_c0, and c43652.graph_c0) in our transcriptome data, but little expression of *VgLPCAT2* genes were detected. *VgLPCAT1* showed high expression during seed development, suggesting its roles in enriching acyl-CoA pool with EFA-CoA. Previous studies showed that PDCT channels PC-derived DAG to TAG in some oilseeds. Overexpression of castor bean *PDCT* gene enhanced novel product levels in hydroxy fatty acid-producing Arabidopsis seeds [15]. PDCT also regulated the polyunsaturated fatty acid accumulation in Arabidopsis [33]. However, the expression level of *VgPDCT* genes was nearly non-detectable in *V. galamensis* seeds. Similar to the case reported here, not any transcripts for PDCT were detected in transcriptomic analysis of bitter melon seeds, which produce high levels of eleostearic acid, an unusual conjugated fatty acid [27]. These suggested that *PDCT* genes might play a minor role in unusual fatty acid accumulation, at least for EFA and conjugated fatty acid. In the subsequent tobacco leaf transient assay (Table 1), VgLPCAT1 and VgPDCT showed distinctive roles in the selective accumulation of EFA, which was consistent with their different expression patterns.

As important enzymes catalyzing the final step in TAG formation, both *VgDGAT* and *VgPDAT* genes had high expression levels in *V. galamensis* seed (Fig. 4) and thus appeared to contribute to EFA accumulation. In plants, these two types of enzymes are involved in the different routines. Generally, PDAT produces the TAG by transferring *sn*-2 FA from PC into *sn*-3 position of DAG, while DGAT converts fatty acid from acyl-CoA to the *sn*-3 position of diacylglycerol (DAG) in Kennedy pathway. In Arabidopsis, PDAT and DGAT1 were major enzymes with overlapping functions for catalyzing TAG production, while the function of DGAT2 remained unclear [34]. In plants accumulating unusual fatty acids such as *Ricinus communis* and *Vernicia fordii*, DGAT2 genes were more highly expressed than DGAT1, thus DGAT2 was proposed to be important for incorporation of unusual fatty acids into TAGs [35, 36]. DGAT2 was also abundant in olive and oil palm for accumulating common fatty acids [37, 38]. These results indicated that different plant species might have different routes to produce TAGs. Although Yu et al. have showed that *VgDGATs* have higher specificity for acyl substrates containing EFA [12], we found DGAT1, DGAT2 and PDAT all contributed to the EFA enrichment, with VgPDAT as the major player. In general, the high level of EFA incorporated into TAG molecules is usually dependent not only on the active synthesis of EFA, but also on the efficient assembly mechanism for the incorporation of EFA into TAGs.

Similar to the reports in other oilseeds, many oleosins, caleosins and steroleosins with very high transcripts were detected in *V. galamensis* seeds [39]. These proteins provided a central hydrophobic region, being capable of protecting TAGs in the form of oil bodies. Considering the sequence variability of amphipathic domain in oleosins from different species, Yang et al. suggested that oleosins may have some substrate specificity [39]. It is possible that one or more of oleosins from *V. galamensis* may specifically package EFA-rich TAG to oil bodies. Notably, three unigenes (c22074.graph_c0, c27706.graph_c0 and c7651.graph_c0) encoding oleosin-like proteins were expressed in exceptional abundance at all three seed development stages. These proteins may be the major oleosin isoforms in oil-body formation of *V. galamensis* seeds.

It was interesting that β-oxidation was active during *V. galamensis* seed development. This pathway was originally upregulated during seed germination, which is a main process to provide essential energy for seedling growth [40]. However, we found that several β-oxidation related genes were highly upregulated during EFA quickly accumulated stage. The activity of β-oxidation was quite distinct from that of FA and TAG biosynthesis stage in other oil seeds, except for the pattern in tung tree, which contained high levels of uncommon eleostearic fatty acids [41]. It is generally known that high concentrations of free unusual fatty acids could be toxic for cellular environment. From an evolutionary point of view, the native unusual fatty acid accumulators must have developed highly efficient mechanisms to package most unusual fatty acids into seed oil bodies, and they may also maintained active pathways for disposal of excess free unusual fatty acids [41]. Our observations regarding up-regulated β-oxidation pathway in the fast oil accumulation stage of developing seeds seem to be paradoxical, since developing seeds are usually active in fatty acid and oil synthesis. However, *V. galamensis* seed might be evolutionarily programmed to activate the β-oxidation pathway to degrade excess free EFAs which are not incorporated into TAGs during rapid oil accumulation period.

Transcription factors (TFs) are key regulators in metabolic networks, in which one TF can simultaneously regulate the expression of multiple genes, while one gene can be simultaneously regulated by multiple TFs [42]. In this study, we annotated many TFs, indicating that *V. galamensis* seed development is controlled by a complex of regulatory network. Previous studies indicated that WRI1, FUS3, and ABI4 were the key TFs controlling fatty acid biosynthesis and oil accumulation in plant seeds [43]. The analysis of *V. galamensis* transcriptome showed that these three TFs had high expression levels. Overexpression of *WRI1* significantly enhanced the seed oil content in transgenic plants. Increasing evidence has showed that WRI1 is a master regulator in controlling the gene expression of lipid genes in the pathway of fatty acid biosynthesis [44]. Notably, we found that WRI1 is important in the early and middle period of *V. galamensis* oil accumulation, as reported for Arabidopsis [45]. Studies have also revealed that both FUS3 and ABI4 are involved in direct or indirect regulation of the fatty acid biosynthesis and TAG accumulation in other plants [46, 47]. These two TFs may promote oil accumulation in the late period of *V. galamensis* lipid synthesis. However, we did not detect DEGs for other lipid-regulated TFs, such as LEC1, LEC2, and MYB89 in any stages tested, suggesting more efforts should be paid to enlarge the pool of transcription factors for this species in future studies.

Remarkably, we employed the transient expression assay in tobacco leaves evidenced that a set of genes identified from this transcriptome function importantly in EFA biosynthesis and EFA-enriched TAG accumulation, including *VgFAD2-like*, *VgLPAT*, *VgDGAT* and *VgPDAT*. Regarding *FAD2-like* gene, it should be noted that some reports showed that epoxygenase (FAD2-like) derived from other plants could catalyze both 18:2 and 18:3 substrates to generate 18:1E (vernonic acid) and 18:2E (12,13-epoxy-*cis*-9,15-octadec-2-enoic acid) [5, 11]. Our data showed that VgFAD2-like is an epoxygenase which mainly catalyzes the synthesis of 18:1E. The transient expression assay of *VgFAD2-like* gene revealed that no 18:2E was detected in tobacco leaves of the target gene transient expression. Furthermore, the full-length cDNA of *VgFAD2-like* was cloned into the yeast expression vector pYES2.0, which was subsequently introduced into yeast (*Saccharomyces cerevisiae*) strain INVSc1N. The transgenic yeast was cultured in the medium with addition of exogenous C18:2 or C18:3. As a result (Additional file 13), under normal growth conditions (30 ℃), the yeast cells carrying *VgFAD2-like* produced an additional peak when cultured in the medium supplemented with C18:2. This peak was identified as 18:1E. Whereas, when cultured in the medium supplemented with C18:3, the transgenic yeast cells produced another peak identified as 18:2E, suggesting that VgFAD2-like may also catalyze C18:3 substrate to form 18:2E in the yeast. Such different epoxy products generated by VgFAD2-like may be related to host cells and the amount of substrates. Further studies are needed to examine the enzymatic characteristics of VgFAD2-like and other EFA-related enzymes.

## Conclusion

*Vernonia galamensis* seed is valued for its high content of natural EFA. Transcriptome sequencing from developing *V. galamensis* seeds and one mixed sample was carried out to identify candidate genes responsible for the biosynthesis and accumulation of EFA-enriched oil. A total of 67,114 unigenes with an N50 length of 1316 nt were assembled from 65 million raw cDNA reads, and 53.16% of them had homology with known genes. Focused on EFA biosynthesis and EFA-enriched TAG assembly, we have identified nearly all of the known genes for de novo EFA biosynthesis, exportation from the plastid, TAG assembly and storage, including Kennedy pathway and PC-mediated TAG synthesis, and TAG degradation. By analyzing the expression profiles, we found that the genes associated with acyl editing, fatty acid β-oxidation, TAG assembly and oil body formation had greater expression levels at the mid-development stage (38 DAP), which are consistent with the fast phase of EFA accumulation in *V. galamensis* seeds, indicating their crucial roles in EFA production. The transient assay of selected genes resulted in a synergistic increase of EFA-enriched TAG accumulation in tobacco leaves. Moreover, we isolated some transcription factors (such as WRI1, FUS3 and ABI4) which putatively regulated the *V. galamensi*s seed oils enriched in EFA. The sequence and gene expression data presented here will enrich our understanding of EFA biosynthesis and regulation in *V. galamensis* seed, providing a set of target genes for commercial production of natural epoxy oil in the established oilseed crops or high-biomass plants by gene engineering.

## Supplementary Information

**Additional file 1: Table S1.** Gene sequences used for transient expression in *N. benthamiana*.

**Additional file 2: Table S2.** Gene-specific primer sequences for detection by qRT-PCR.

**Additional file 3: Table S3.** Statistics of *V. galamensis* sequencing data.

**Additional file 4: Table S4.** Summary details of assembled sequences produced by Trinity.

**Additional file 5: Table S5.** Summary of functional annotations.

**Additional file 6: Figure S1.** Functional annotation and classification of unigenes in *V. galamensis*. (a) Overall classification of 21,364 unigenes distributed into major GO categories. (b) Eukaryotic Orthologous Groups (KOG) analysis of 28,896 unigenes which were classified into 26 functional groups.

**Additional file 7: Table S6.** 8371 unigenes mapped to EC numbers in 119 KEGG pathways.

**Additional file 8: Figure S2.** Clustering diagram of enriched GO terms from 651 annotated differentially expressed genes (DEGs). Genes were assigned into three main categories: biological processes, cellular components or molecular functions.

**Additional file 9: Table S7.** Significantly enriched GO terms from differentially expressed genes.

**Additional file 10: Table S8.** List of genes involved in EFA metabolism in *V. galamensis* seed.

**Additional file 11: Table S9.** FPKM values for fatty acid β-oxidation related genes.

**Additional file 12: Table S10.** Correlation coefficients between qRT-PCR and RNA-Seq results for selected 12 genes.

**Additional file 13: Table S11.** Fatty acid composition in *Saccharomyces cerevisiae* cells overexpressing *VgFAD2-like* when cultured in the medium with addition of exogenous C18:2 or C18:3.

## Data Availability

The data sets generated and/or analyzed during the current study are included in Additional Tables.

## Abbreviations

ACCase: Acetyl-CoA carboxylase
ACP: Acyl carrier protein
ACX: Acyl-CoA oxidase
BC: Biotin carboxylase
BCCP: Biotin carboxyl carrier protein
DAP: Days after pollination
DEG: Differentially expressed gene
DGAT: Acyl-CoA:diacylglycerol acyltransferase
EAR: Enoyl-ACP reductase
ECH: Enoyl-CoA hydratase
EFA: Epoxy fatty acid
FA: Fatty acid
FAD2: Δ12-Fatty acid desaturase
FAD2-like: Δ12-Fatty acid epoxygenase
FAD3: Δ15-Fatty acid desaturase
FAME: Fatty acid methyl ester
Fat: Acyl-ACP thioesterase
FPKM: Fragments Per Kilobase of transcript per Million fragments mapped
FUS3: FUSCA3
GO: Gene ontology
HAD: 3-Hydroxyacyl-ACP dehydratase
KAR: NADPH-dependent beta-ketoacyl-ACP reductase
KAS: Beta-ketoacyl-ACP synthase
KAT: 3-Ketoacyl-CoA thiolase
KOG: Eukaryotic Orthologous Groups
KEGG: Kyoto Encyclopedia of Genes and Genomes
LACS: Long chain acyl-CoA synthetase
LEC1: LEAFY COTYLEDON 1
LPCAT: Lysophosphatidylcholine acyltransferase
MCAT: Malonyl-CoA:ACP transacylase
Nr: NCBI non-redundant database
PC: Phosphatidylcholine
PDAT: Phospholipid:diacylglycerol acyltransferase
PDCT: Phosphatidylcholine:diacylglycerol cholinephosphotransferase
SAD: Stearoyl-ACP desaturase
TAG: Triacylglycerol
α-CT: Alpha-carboxyltransferase
β-CT: Beta-carboxyltransferase
