# Supplementary figures and images for: Critical metabolic pathways and genes cooperate for epoxy fatty acid-enriched oil production in developing seeds of Vernonia galamensis, an industrial oleaginous plant
Source: Biotechnol Biofuels Bioprod. 2022 Feb 25;15:21. doi: 10.1186/s13068-022-02120-2 (PMC8881847; doi:10.1186/s13068-022-02120-2)

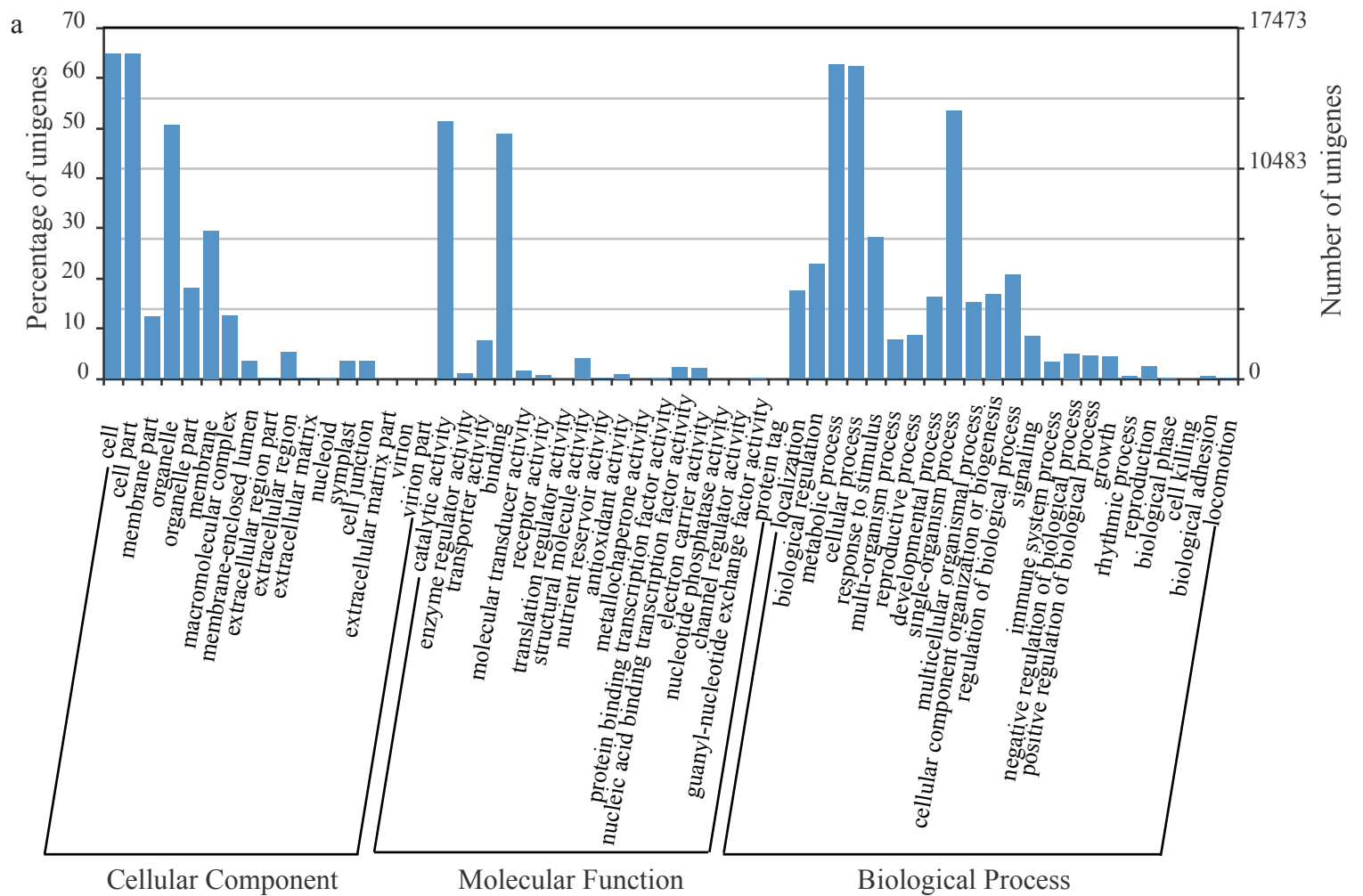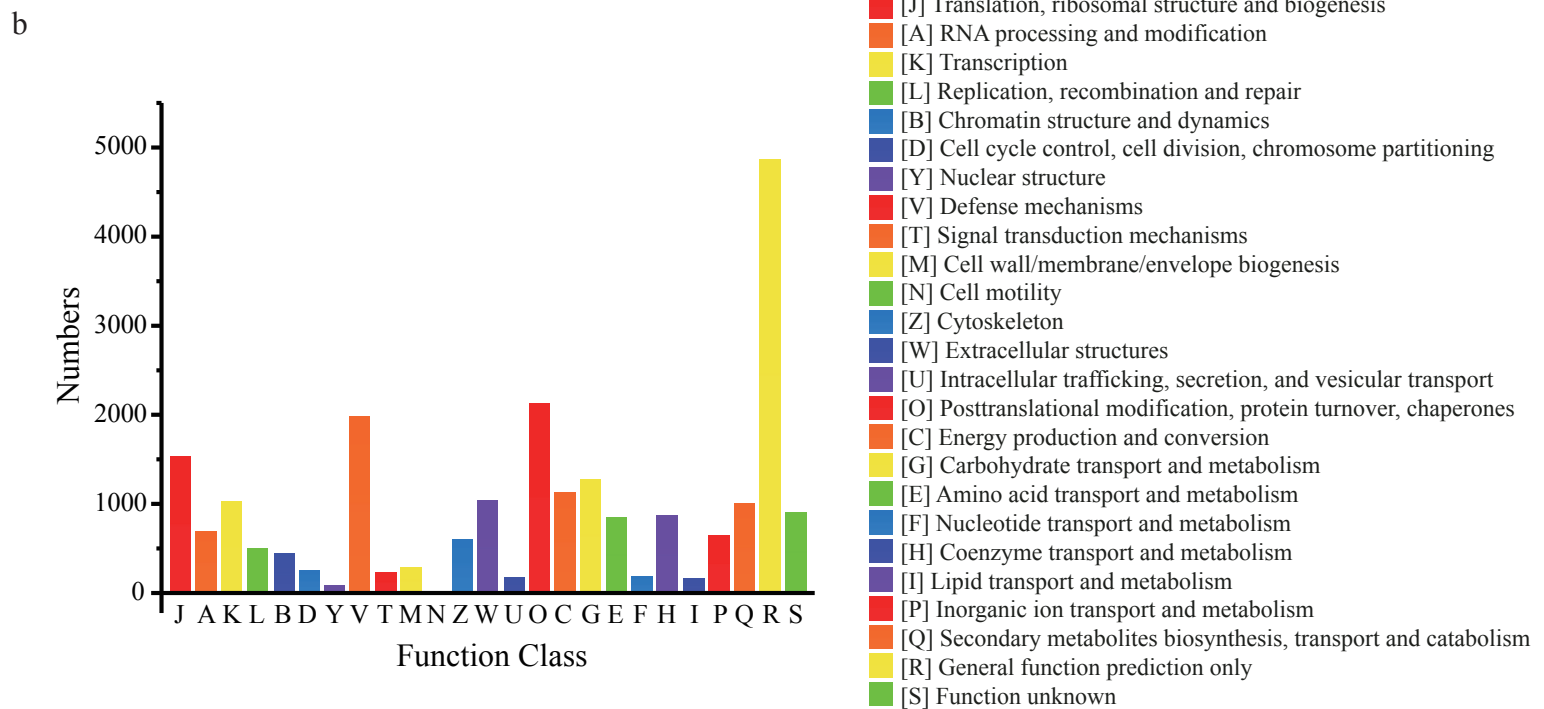

Supplement: Supplementary file 6 — Additional file 6: Figure S1. Functional annotation and classification of unigenes in V. galamensis. (a) Overall classification of 21,364 unigenes distributed into major GO categories. (b) Eukaryotic Orthologous Groups (KOG) analysis of 28,896 unigenes which were classified into 26 functional groups. [file 13068_2022_2120_MOESM6_ESM.pdf]

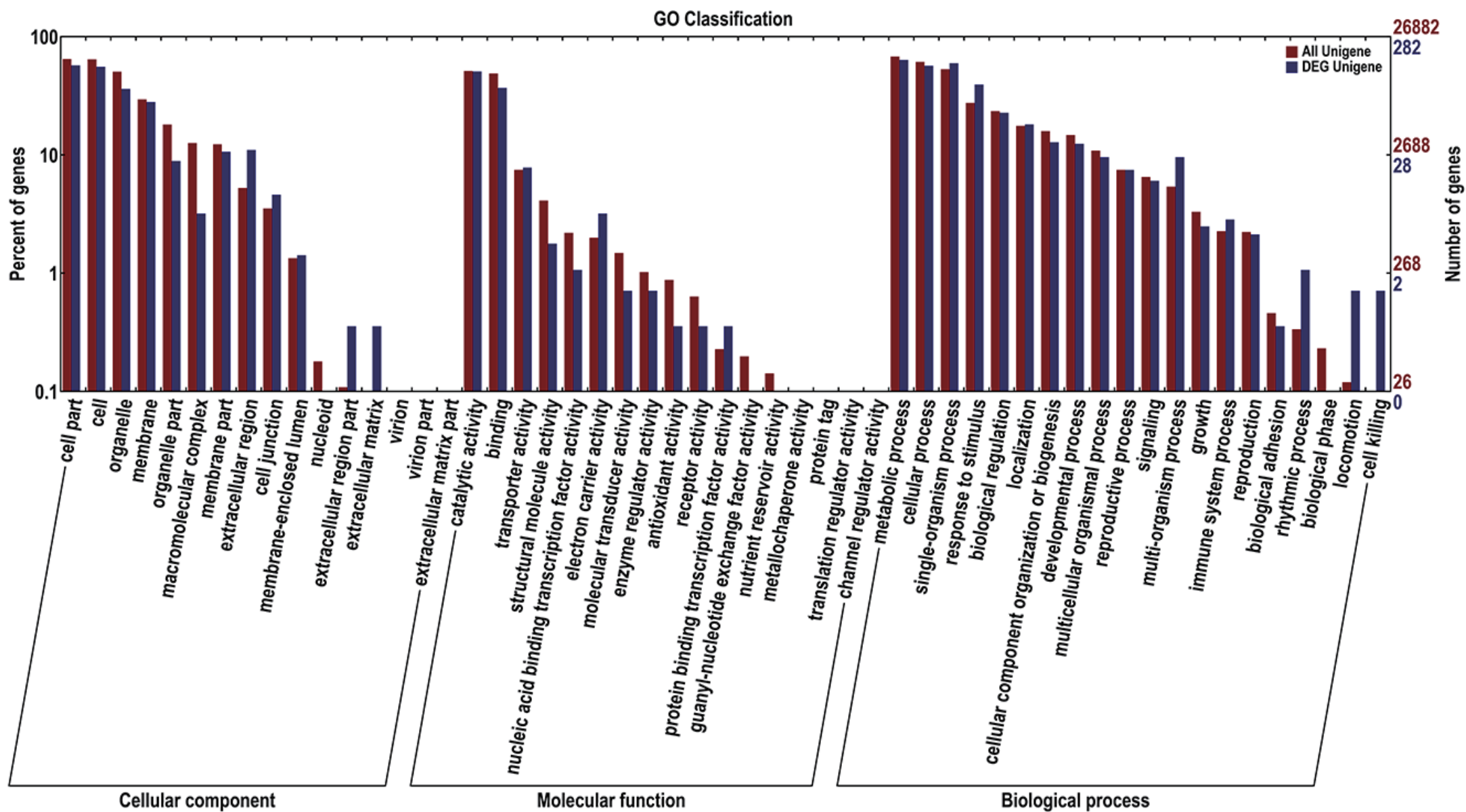

Supplement: Supplementary file 8 — Additional file 8: Figure S2. Clustering diagram of enriched GO terms from 651 annotated differentially expressed genes (DEGs). Genes were assigned into three main categories: biological processes, cellular components or molecular functions. [file 13068_2022_2120_MOESM8_ESM.pdf]
